# CRE/CREB-Driven Up-Regulation of Gene Expression by Chronic Social Stress in CRE-Luciferase Transgenic Mice: Reversal by Antidepressant Treatment

**DOI:** 10.1371/journal.pone.0000431

**Published:** 2007-05-09

**Authors:** Ulrike Böer, Tahseen Alejel, Stephan Beimesche, Irmgard Cierny, Doris Krause, Willhart Knepel, Gabriele Flügge

**Affiliations:** 1 Department of Molecular Pharmacology, University of Göttingen, Göttingen, Germany; 2 Laboratory of Clinical Neurobiology, German Primate Center, Göttingen, Germany; 3 DFG Research Center Molecular Physiology of the Brain (CMPB), Göttingen, Germany; The Babraham Institute, United Kingdom

## Abstract

**Background:**

It has been suggested that stress provokes neuropathological changes and may thus contribute to the precipitation of affective disorders such as depression. Likewise, the pharmacological therapy of depression requires chronic treatment and is thought to induce a positive neuronal adaptation, presumably based on changes in gene transcription. The transcription factor cAMP-responsive element binding protein (CREB) and its binding site (CRE) have been suggested to play a major role in both the development of depression and antidepressive therapy.

**Methodology/Principle Findings:**

To investigate the impact of stress and antidepressant treatment on CRE/CREB transcriptional activity, we generated a transgenic mouse line in which expression of the luciferase reporter gene is controlled by four copies of CRE. In this transgene, luciferase enzyme activity and protein were detected throughout the brain, e.g., in the hippocampal formation. Chronic social stress significantly increased (by 45 to 120%) CRE/CREB-driven gene expression measured as luciferase activity in several brain regions. This was also reflected by increased CREB-phosphorylation determined by immunoblotting. Treatment of the stressed mice with the antidepressant imipramine normalized luciferase expression to control levels in all brain regions and likewise reduced CREB-phosphorylation. In non-stressed animals, chronic (21 d) but not acute (24 h) treatment with imipramine (2×10 mg/kg/d) reduced luciferase expression in the hippocampus by 40–50%.

**Conclusions/Significance:**

Our results emphasize a role of CREB in stress-regulated gene expression and support the view that the therapeutic actions of antidepressants are mediated via CRE/CREB-directed transcription.

## Introduction

Depression is a prevalent mood disorder with a high incidence. Though the etiology of depressive diseases is not yet fully understood, there is evidence that besides genetic factors[1] also environmental influences such as stressful life events are involved in its development[2]. Chronic stress has multiple effects on the organism. In addition to immediate neuroendocrine responses such as the activation of the hypothalamic-pituitary-adrenal axis [2], [3] and up-regulation of the sympathetic nervous system activity[4], chronic stress induces changes in gene expression [5], [6]. These changes can account for long-term effects in the nervous system that are considered to contribute to the pathophysiology of depression[7].

Antidepressant drugs are widely used to treat depressive mood disorders since the 1950s. Although their effectiveness is undisputable, the mechanisms of their action are still not completely understood. As antidepressants increase the extracellular concentration of monoamines through inhibition of reuptake or degradation, they were thought to correct monoamine deficiency that has been implicated in pathogenesis of depression[8]. However, there is a clear discrepancy between antidepressants rapidly increasing extracellular monoamine concentrations and the lack of immediate clinical efficacy in that antidepressive effects usually occur only with a delay of two to three weeks. This lag phase has been considered to be related to drug-induced neuronal plasticity and may be explained at the molecular level by changes in gene expression[6].

Among the proteins that regulate gene transcription the transcription factor CREB (cAMP responsive element binding protein) is one of the most widely expressed. CREB belongs to the family of basic leucine zipper transcription factors and modulates gene transcription by binding to the cAMP responsive element (CRE) in the promoter regions of various genes[9]. In the central nervous system CREB action was first described for the adrenergic system where generation of cAMP by β-adrenergic receptors activates protein kinase A which in turn phosphorylates CREB at serine 133 and thereby induces gene transcription[10]. Nowadays, several signalling cascades including a large number of receptors and kinases are known to converge at the level of CREB, thus underlining its importance for neuronal activity[11]. CREB activity has been particularly implicated in neuro-adaptative processes such as learning and memory [12], [13] and addiction[14].

In the past, several studies tried to determine the role of CREB in antidepressant action the majority of which reported on CREB phosphorylation as an indicator for its transcriptional activity[15]. However, it has been shown that phosphorylation is a necessary prerequisite for CREB-mediated transcription but not fully sufficient[16]. Therefore CRE/CREB-directed regulation of gene transcription cannot be determined by measuring simply CREB phosphorylation but rather by determining expression of a CRE/CREB-driven gene product which requires the generation of transgenic animals carrying a reporter gene. However, there is only one study in mice investigating effects of an antidepressant on a CRE-driven LacZ reporter[17]. Furthermore, there is currently no information related to the actions of stress on CRE/CREB-directed gene transcription *in vivo*. Therefore, to contribute to the understanding of CREB transcriptional activity *in vivo*, we generated a transgenic mouse line in which the transgene is a CRE-driven luciferase (Luc) reporter gene. This transgenic mouse allows direct qualitative and quantitative monitoring of CRE/CREB transcriptional activity by measuring luciferase enzymatic activity. In the present study we characterize the expression and functionality of the reporter gene in the CRE-Luc transgenic mice. Furthermore, we determined effects of chronic social stress and of the antidepressant imipramine on CRE/CREB-directed gene transcription *in vivo*.

Transgenic mice were submitted to chronic psychosocial stress using a well established experimental paradigm that serves as a model for depression[18]. Our results indicate that CRE/CREB-directed gene transcription is enhanced by chronic psychosocial stress and that treatment with imipramine reverses the stress-induced effect on CREB activation *in vivo*. To our knowledge, this is the first report providing direct evidence that CRE/CREB mediates stress effects on the transcriptional level and that the antidepressant imipramine normalizes stress-elevated CRE/CREB-directed gene transcription *in vivo*.

## Results

### Generation and analysis of transgenic reporter CRE-Luc mice

**Figure 1:**
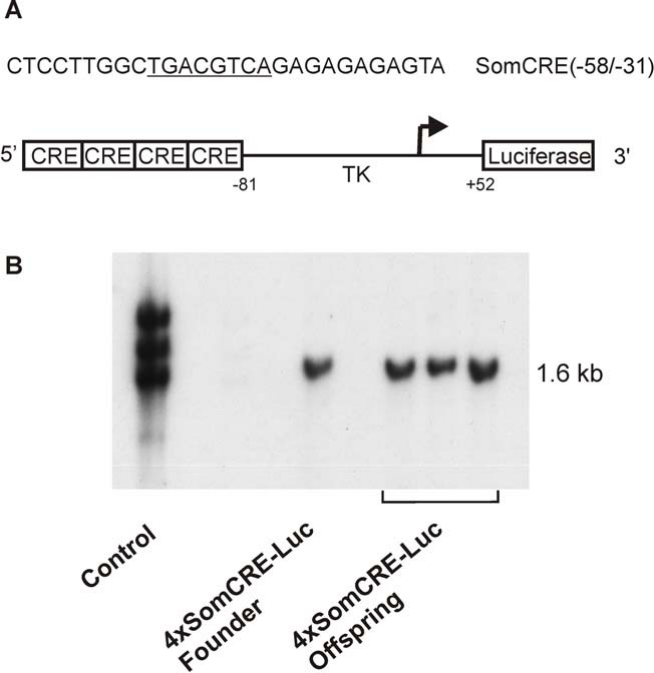
Schematic representation of the DNA construct used to generate transgenic mice. A: Four copies of the rat somatostatin gene promoter CRE (SomCRE) (5′-GATCCTCCTTGGCTGACGTCAGAGAGAGAGTA-3′) were fused with a truncated thymidine kinase promoter (−81 to+52) and the firefly luciferase gene[19]. The CRE is underlined. B: Presence of the CRE-Luc construct in founder and offspring mice was detected by Southern blotting using genomic DNA from tail biopsy. DNA was digested with *Bgl*II, electrophoresed, and immobilized on a Nylon membrane by capillary blotting. DNA was probed with a [α-^32^P]-labeled 1.6-kb *Xba*I luciferase gene fragment. Shown is a typical blot with the 1.6 kb bands indicating the transgene. As control served genomic DNA from a luciferase reporter mouse generated previously[52].

For the generation of transgenic reporter mice 4 copies of the rat somatostatin gene promoter CRE and the truncated thymidine kinase promoter were fused with the firefly luciferase-encoding gene (Fig. 1A). The sequence of the rat somatostatin gene promoter CRE is a well characterized binding site for the transcription factor CREB [19]. The construct was microinjected as linear fragment into the pronuclei of one-cell embryos. Transgenity of the offspring was checked by Southern blot analysis. Hybridization of the radioactively labelled probe revealed a 1.6 kb fragment of the luciferase gene in the genomic DNA (Fig. 1B). From three independently derived transgenic mouse lines one was chosen that had highest luciferase gene expression in the brain.

**Figure 2:**
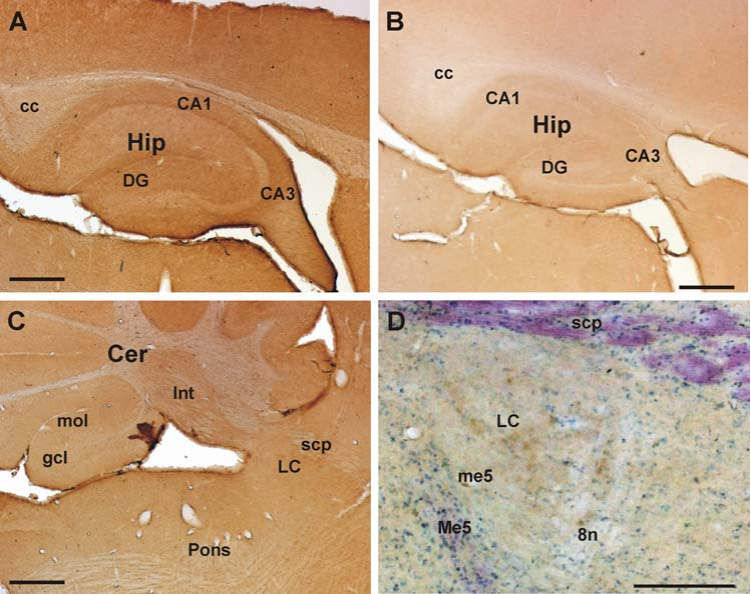
Reporter gene expression in transgenic CRE-Luc mice. Luciferase immunoreactivity in a CRE-Luc (A, C, D) and a control mouse (B). Luciferase antibody binding was visualized by 3,3′-diaminobenzidine staining on sagittal sections (brown reaction product in A to D); the section in D was counter stained with cresyl violet. Note that Luciferase immunoreactivity in the hippocampal formation (Hip) of the transgene mouse (A) is stronger than in the control (B). In the cerebellum (Cer in C), the molecular (mol) and the granule cell layer (gcl) as well the interposed nuclei (int) show moderate immunoreactivity. In the locus coeruleus (LC in D), distinct cells (brown) are moderately stained by the antibody. Abbreviations: cc, corpus callosum; CA1 and CA3, hippocampal regions cornu ammonis 1 and 3; DG, dentate gyrus; scp, superior cerebellar peduncle; Me5, mesencephalic trigeminal nucleus; me5, mesencephalic trigeminal tract; 8n, vestibulocochlear nerve. Scale bars: 500 µm (in A, B, C) and 200 µm (in D).

Luciferase protein was determined by immunocytochemistry with specific antibodies. Throughout the brain sections, transgenic mice showed a diffuse brown staining which was stronger than in control animals (Fig. 2). In the hippocampal formation, regions CA1, CA3 and dentate gyrus were labelled indicating luciferase expression in pyramidal and granule cell neurons as well as other cells (Fig. 2A). In the cerebellum, the antibody equally labelled the molecular and the granule cell layer and interposed nuclei (Fig. 2C). Also pons and brain stem displayed an almost homogeneous staining pattern. Furthermore, in the area of the locus coeruleus brown patches indicated staining of large neurons (Fig. 2D). Similarly, in regions of the brain stem large neurons were intensively stained which could be identified as pontine motoneurons (not shown). Thus, luciferase protein appears to be localized ubiquitously in brain cells including neuronal expression.

**Figure 3:**
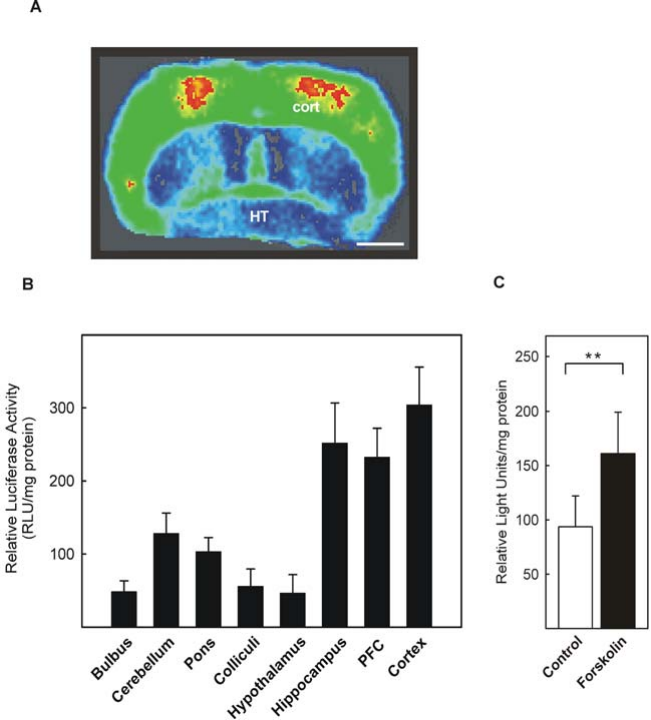
Reporter gene expression in CRE-Luc mice. A: Imaging of luciferase enzyme activity. A coronal brain section was covered with the substrate luciferin and emitted light was visualized with a slow scan CCD camera system (pseudocolour image with red representing strong and blue representing low enzymatic activity). Luciferase activity was detected predominantly in cortex (cort) and to lesser extend in the hypothalamus (HT). Shown is a representative section on a level with the optic chiasm. Scale bar: 2 mm. B: Quantification of luciferase expression in different brain regions. Luciferase enzyme activity was measured in homogenates of different brain regions and normalized to protein concentrations. Highest enzyme activity was detected in cortex, hippocampus and prefrontal cortex (PFC). Values are means±SEM (standard error of the mean) from 6 animals. C. Forskolin-induced reporter gene expression. Coronal vibratome sections of fresh brains (interaural from 0–6) from CRE-Luc mice were incubated with 10 µM forskolin for 6 h and luciferase activity was determined. Forskolin treatment resulted in a 1.93 fold increase of luciferase activity (means±SEM from 7 independent experiments). Significant difference **: p<0.01; Wilcoxon test for paired samples.

To determine luciferase enzyme activity cryostat sections from CRE-Luc mice were covered with luciferin substrate solution and imaged by a CCD camera system (Fig. 3A). A pronounced luciferase activity was detected in the cortex. To quantify the luciferase activity different brain regions were dissected and enzyme activity was measured using a luminometer (Fig. 3B). Luciferase activity was most intensive in cortex (303 RLU/mg protein), hippocampus (252 RLU/mg protein) and PFC (232 RLU/mg protein) followed by cerebellum (128 RLU/mg protein), pons (103 RLU/mg protein), colliculi (56 RLU/mg protein), bulbus olfactorius (49 RLU/mg protein) and hypothalamus (47 RLU/mg protein). Eyes showed only poor luciferase activity (3 RLU/mg protein), therefore eyes were excluded from further experiments.

Phosphorylation of CREB is a necessary step in the activation of transcription. To determine basal CREB phosphorylation in the respective brain regions, western blot analysis was performed using CREB and phospho-CREB specific antibodies. The basal phospho-CREB/CREB ratio was 0.22±0.023 in bulbus olfactorius, 0.29±0.034 in cerebellum, 0.32±0.008 in pons, 0.32±0.046 in colliculi, 0.22±0.089 in PFC, 0.25±0.073 in hypothalamus, 0.26±0.095 in cortex and 0.26±0.064 in hippocampus. Thus, basal CREB phosphorylation was constant over the different regions.

To ensure that the promoter was still able to confer stimulus-dependent luciferase gene transcription, vibratome slices of CRE-Luc mice were incubated with 10 µM forskolin to induce CRE/CREB-directed transcription (Fig. 3C). The adenylate cyclase activator forskolin increased luciferase activity to 191% of control thus showing that the promoter construct was intact and inducible.

### Chronic psychosocial stress

As stress has been implicated in the development of depression we determined the effect of chronic psychosocial stress on the CRE/CREB-directed transcription. The sensory contact model provides a useful tool for this purpose. In this model the subordinate mouse is chronically stressed by the unavoidable sensory contact with the dominant conspecific. Stress-specific behaviour such as chasing and biting in dominants and flying and squeaking in subordinate animals was clearly identified by two independent investigators. In addition, reduced body weight gain in the subordinate animals indicated that these animals experienced stress. After 21 days, the body weight gain of subordinate animals was reduced to 96% of the value for the first day thus differing significantly from weight gain in controls (Table 1).

**Table 1:** Body weight gain in control and subordinate CRE-Luc mice.

Body weight gain (%)					
Day	Control	Stress	Day	Control (solvent)	Stress+Imipramine
1	100-	100-	1	100-	100-
4	101.74±0.85	99.57±0.31	6	100.87±1.04	103.31±0.12
7	101.04±1.16	97.26±0.58 *	10	99.05±1.93	104.52±0.04*
11	101.42±1.42	97.08±1.02 *	17	98.62±2.25	104.50±0.06*
14	98.89±0.93	95.90±0.93	20	98.72±2.26	104.04±0.07
21	100.18±0.64	96.17±0.64 **	25	99.27±2.88	105.80±0.07**

Body weight of control and subordinate mice was determined on different days of the experimental time period and expressed as percentage of the mean value of the same group on day 1. Values are percentage of the mean value in controls (±SEM) from 5 (control, untreated littermates), 7 (stress), 6 (control, solvent-treated littermates) and 6 (stress+imipramine) animals per group.

**Figure 4:**
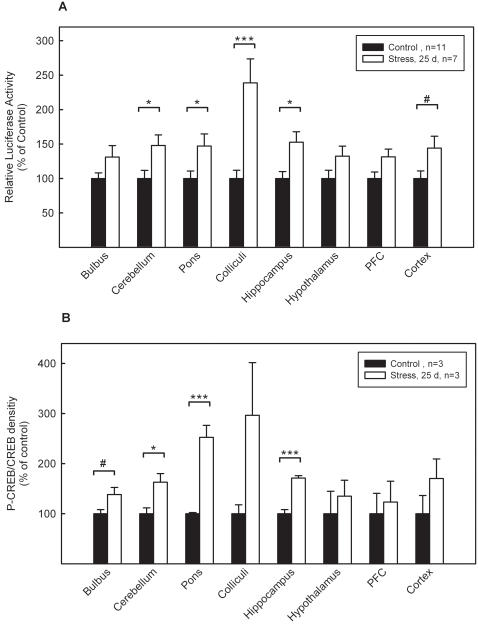
Effect of chronic social stress on reporter gene expression. Male CRE-Luc mice were either not treated (open bars) or exposed daily to social stress for 25 days (black bars). A: Luciferase activity was measured in homogenates of different brain regions, normalized to protein contents and expressed as percentage of the mean value in controls (unstressed littermates) (±SEM from 7 animals). Stress increased luciferase activity in all brain regions reaching statistical significance in cerebellum, pons, colliculi and hippocampus. Significant differences *, p<0.05; ***, p<0.001; #, p = 0.059 as determined by two-way ANOVA followed by Duncan's *post hoc* test. B: Quantification of CREB and phospho-CREB levels by western blot analysis. Homogenates of the respective regions were submitted to immunoblotting using specific CREB and phospho-CREB antibodies. Intensity of specific bands was determined densitrometrically and ratios between CREB and phospho-CREB were calculated. Unstressed littermates served as controls. Values are means±SEM from 3 animals. Significant differences *, p<0.05; ***, p<0.001; #, p = 0.078 as determined by two-way ANOVA followed by Duncan's *post hoc* test. PFC: prefrontal cortex

The impact of stress on the CRE/CREB-directed transcription in the brain of the subordinate mice was determined by measuring reporter gene expression. Brain regions were dissected and luciferase activity was measured. Animals kept without stress in a familiar environment served as controls. Chronic psychosocial stress increased luciferase activity significantly in cerebellum (147.9%), pons (147.2%), colliculi (238.9%) and hippocampus (152.7%). In cortex (144.1%) values only approached significance (p = 0.059) (Fig.4A). Bulbus, hypothalamus and PFC showed a tendency to increased luciferase activity.

To examine CREB phosphorylation in mice subjected to chronic stress throughout the brain, the dissected regions were analyzed by immunoblotting. Each brain region tested showed a tendency for elevated CREB phosphorylation after chronic stress which reflected the increase in luciferase activity. Values reached significance in cerebellum (163%), pons (252%) and hippocampus (171%) (Fig.4B). The mean increase in CREB phosphorylation over all regions was 181%.

### Acute and chronic treatment with imipramine

To test the action of antidepressants on CRE/CREB-directed transcription CRE-Luc mice were treated acutely (24 h) and chronically (21 days) with imipramine (Table 2). Acute treatment did not result in significant changes in luciferase activity in any brain region though there was a slight tendency towards an increase. In contrast, chronic imipramine caused a decrease in luciferase activity (Table 2). Luciferase activity was significantly reduced in the hippocampus (to 48.3%) whereas the other regions showed only a tendency for diminished luciferase activity.

**Table 2:** Luciferase activity in imipramine-treated CRE-Luc mice.

	Acute treatment (24 h)		Chronic treatment (21 d)	
	Control	Imipramine	Control	Imipramine
Bulbus	100±20.9	156.6±16.0	100±9.5	78.0±25.0
Cerebellum	100±33.6	162.7±31.5	100±9.3	100.2±15.0
Pons	100±34.2	142.3±50.9	100±11.2	70.2±9.1
Colliculi	100±53.0	197.1±53.9	100±8.6	80.8±10.9
Hippocampus	100±25.5	115.3±31.4	100±20.6	48.4±6.3*
Hypothalamus	100±34.7	113.4±25.5	100±20.1	85.0±18.6
PFC	100±33.9	82.2±31.2	100±9.7	83.8±9.3
Cortex	100±29.1	105.0±36.1	100±11.8	74.5±9.5

Acute and chronic treatment with imipramine was performed in CRE-Luc mice that were not stressed. PFC: prefrontal cortex. Values are percentage of the mean value in controls (solvent-treated littermates) (±SEM) from 3 (acute) and 12 (chronic) animals per group.

### Imipramine treatment in chronically stressed transgenic mice

**Figure 5:**
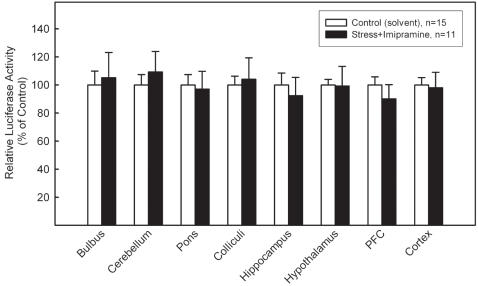
Effect of imipramine on the stress-induced increase in CRE/CREB-directed luciferase expression. Male CRE-Luc mice were either solvent treated (white bars) or exposed to chronic social stress for 25 days and treated with imipramine for 21 days (black bars) while the daily stress exposure continued. Luciferase activity was measured in homogenates of different brain regions and normalized to protein contents and expressed as percentage of the mean value in controls (solvent-treated littermates) (±SEM from 9 animals). In all brain regions, imipramine reversed the stress-induced increase in luciferase activity resulting in data close to control. PFC: prefrontal cortex

To investigate the influence of chronic imipramine on expression of the luciferase reporter gene in stressed CRE-Luc mice, subordinate mice were treated for 21 days with imipramine while the daily social stress exposure continued. Luciferase activity in the brain was measured at the end of the experiment. Under these conditions luciferase activity in all brain regions tested did not differ significantly from control levels indicating that the antidepressant normalized enzyme expression in the stressed animals (Fig. 5). Thus, the chronic treatment with imipramine completely abolished the stimulating effect of chronic social stress on CRE/CREB-directed transcription and resulted in levels similar to control.

### Effect of imipramine treatment on the stress-induced increase in CREB-phosphorylation

**Figure 6:**
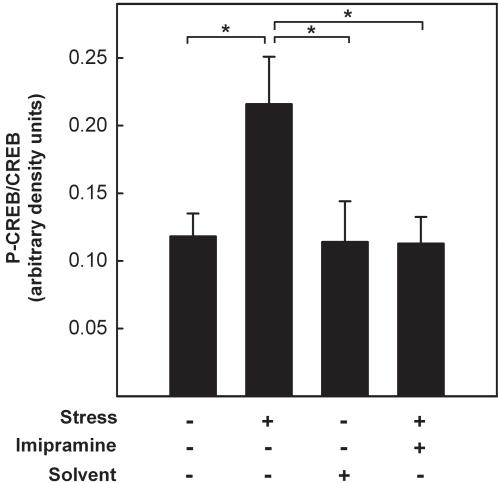
Effect of imipramine on stress-induced CREB phosphorylation. Male CRE-Luc mice were exposed to chronic psychosocial stress with or without concurrent imipramine treatment for 21 days. Homogenates of whole brains were submitted to immunoblotting using specific CREB and phospho-CREB antibodies. Intensity of specific bands was determined densitrometrically and ratios between CREB and phospho-CREB were calculated. Solvent-treated littermates served as controls. Values are means±SEM from 5 animals. Significant difference: *:p<0.05; Kruskal-Wallis test followed by Schaich Hamerle-*post hoc* test.

To examine whether imipramine also has an effect on the stress-induced phosphorylation of CREB we determined CREB phosphorylation in mice subjected to chronic stress plus imipramine treatment. Here, whole brain lysates of the respective mice were subjected to western blot analysis. Chronic stress resulted in an 1,8-fold increase of the ratio phospho-CREB/CREB compared to control (Fig. 6) which corresponds exactly with the calculated mean increase of the distinct regions. In line with the luciferase data, treatment with imipramine during chronic social stress reduced CREB phosphorylation to control levels. Thus, imipramine treatment does not only reverse stress-induced increase of luciferase enzyme activity but also normalizes CREB phosphorylation.

## Discussion

In this study we determined the effect of chronic psychosocial stress and of imipramine treatment on the transcriptional activity of CREB *in vivo*. For this purpose a transgenic mouse line was used that carries a CRE-driven luciferase reporter gene as transgene. In previous studies this construct has been characterized thoroughly with respect to its CREB binding affinity and its response to stimuli like cAMP and membrane depolarization[19].

The CRE-driven luciferase reporter gene is a helpful tool to determine transcription. CREB activity cannot be equated with its phosphorylation per se although phosphorylation is a necessary prerequisite for CREB activity as it enables the interaction with the cofactor CBP (CREB binding protein)[9]. CBP has been considered for long time as the only interacting partner of CREB as it conveys activation of CREB to the basal transcription machinery [9]. Recently a second cofactor, TORC, has been discovered which is also crucial for CREB activity but acts independently from phosphorylation of Ser-133[20]. In addition, the immunosuppressant cyclosporine A inhibits CREB transactivational activity despite of its phosphorylation of Ser-133[16]. This supports the notion that for comprehensive characterization of an effect on CREB activity it is not sufficient to determine only phosphorylation of CREB but rather its impact on gene expression. Among the reporter genes used for this purpose the gene coding for the light generating enzyme luciferase combines high sensitivity with dose-response-linearity over a broad concentration range and thus is most suitable for quantitative assessment of promoter activation by CREB.

In the transgenic mouse line the presence of the luciferase gene and its respective product was tested by different approaches. Satisfactory levels of DNA, RNA and protein were detected thus indicating that the reporter gene was expressed. Furthermore, the distribution of the luciferase protein detected by immunohistochemistry indicates that the expression also includes neurons as indicated by labelling of large cells in the locus coeruleus. Basal luciferase activity was relatively low in most brain regions except cortical regions and hippocampus. As CREB functioning has been implicated in neuronal activity[21], basal CREB activity may reflect the level of activated neurons in the mouse brain under unstimulated conditions. The basal CREB phosphorylation did not correlate with the luciferase activity. This discrepancy between CREB phosphorylation and transcriptional activity has been reported before in the visual cortex [22] and the hippocampus [23] leading to the assumption, that additional mechanisms regulating CREB activity must be present. Interestingly, Kovacs et al. recently showed the involvement of TORC in the CREB-dependent synaptic plasticity in the hippocampus[24]. As TORC confers CREB activity independent of phosphorylation on Ser-133, the discrepancy between phosphorylation and activity of CREB could be explained, at least for the hippocampus, by the involvement of TORC.

The present experiments were performed with a transgenic line displaying the highest luciferase levels in the brain; however, experiments for chronic imipramine treatment were also performed with a second transgenic line (data not shown). The results were in agreement with those reported in this study thus indicating that the results were not influenced by a position effect of the inserted construct.

The relationship between stressful life events and the occurrence of psychiatric disorder has led to the development of a number of models using acute or chronic stress to induce depressive-like symptoms in animals such as mice[18], tree shrews[25] and rats[26]. In these models, male animals are stressed by a dominant conspecific to induce “emotional” despair rather than physical discomfort. In the present study the sensory contact model was applied to SomCRE-Luc mice in order to investigate the influence of chronic psychosocial stress on the CRE/CREB-directed transcription. The transgenic status of the mice did not induce behavioural changes in the model as similar social interactions as described for mice were observed[27] and also the changes in body weight indicated somatic effects. To our knowledge we are the first who investigated the effect of chronic psychosocial stress on CREB-driven gene expression *in vivo*. This form of stress showed a tendency to increase CRE/CREB-mediated reporter gene expression in all brain regions examined with a significant effect in cerebellum, hippocampus, pons and colliculi. Stress-induced changes in gene expression has been reported before in the hippocampus[6], dorsal raphe nuclei[5] and the locus coeruleus[28], which are both pontine regions. Though gene expression in the colliculi has not been investigated before, there is strong evidence, that colliculi play a pivotal role in stress-induced behaviour[29], which may include also gene expression.

CREB phosphorylation likewise was significantly enhanced in pons and hippocampus and with a strong tendency in the colliculi, which indicates an enhanced signalling cascade leading to kinase activation in these regions. The effects of signalling pathways involving adenylate cyclase and cAMP, Ca^2+^ and different kinases on CREB/CREB-regulated gene transcription are well established[30]. As one important endocrine stress response is the activation of the adrenergic system[4], most likely the protein kinase A predominantly is involved in stress-induced CREB phosphorylation. Thus, in contrast to the basal CREB activity, under stress there seems to be a strong phosphorylation-dependent regulation. Among the genes being up-regulated by chronic social stress is also CBP[5]. As CBP is a crucial cofactor in mediating phosphorylation-dependent CREB activity, enhanced levels of CBP might reflect increased CREB phosphorylation.

Both findings, CRE/CREB-mediated gene expression and phosphorylation suggest a role of CREB in neuronal adaptation based on changes in gene expression that have been linked to long-term psychosocial stress conditions and cause the basis for the development of depressive disorders[7]. What could be the consequences of up-regulation of CREB activity? One target gene of CREB is the transcription factor c-fos. Indeed, Matsuda et al.[31] could show persistent up-regulated c-fos mRNA levels after chronic psychosocial stress in widespread brain areas including hippocampus, pontine nuclei and colliculi. The authors link c-fos expression to stress-induced gene transcription and neuroplasticity. Also the corticotropin releasing hormone (CRH) gene is regulated by CREB. Stress-induced CREB activity thus could contribute to up-regulation of the hypothalamic-pituitary-adrenal (HPA) axis, which results in elevated glucocorticoid levels[3]. Another target gene of CREB is the brain-derived neurotrophic factor BDNF which is also involved in neuroplasticity. Recently, it was shown, that BDNF in the mesolimbic dopamine pathway[32] and in the hippocampus[33] is upregulated under social defeat stress. However, BDNF also is downregulated by specific stressors[34] and might exert antidepressant actions[35]. These divergent findings parallel the complexitiy of the role of CREB in stress. Other stress paradigms than social defeat resulted in opposite effects on CREB activity, e.g. as consequence of a mild stressor (foot shocks for 21 days) phospho-CREB levels in the hippocampus were reduced[36]. Also variable unpredictable stress (footshock, cold, restraint) decreased phosphorylation of CREB in frontal cortex, hippocampus and striatum[37]. Thus, changes in the transcriptional activity of CREB and of its gene product BDNF depend not only on the brain region but also on the type of stress and its duration.

Furthermore, we examined the action of the antidepressant imipramine on CRE/CREB-directed transcription. Chronic but not acute treatment with imipramine reduced CREB activity which was most pronounced in the hippocampus. Finally, treatment with imipramine of the stressed subordinate mice in the sensory contact model resulted in a reversal of the stress-induced increase in CREB activity to control levels, and also CREB phosphorylation was normalized. These results implicate that chronic psychosocial stress and chronic treatment with the antidepressant imipramine exert opposing effects on CREB.

There are many investigations on the effects of various antidepressants on CREB. Several studies examining the consequences of imipramine reported on inhibitory effects on CREB expression [38], [39] or CREB-regulated transcription[40]. In contrast, Nibuya et al.[41] found an increased CREB expression after chronic imipramine. These data are at variance with our data and those mentioned before. However, the activity of CREB, e.g. its phosphorylation, or its effects in transactivating transcription due to imipramine treatment was not determined in this study. Thus, the in vivo consequences of increased CREB expression so far appear not consistent. Imipramine as a monoamine reuptake inhibitor has a slightly higher affinity for the noradrenaline transporter compared to the serotonin transporter [42]. Interestingly, as reviewed by Tardito et al.[15] particularly the specific pro-noradrenergic antidepressants such as desimipramine and reboxetine have been shown to inhibit CREB activity[43], whereas selective serotonin reuptake inhibitors (SSRI) induce an activation of CREB. Using a transgenic LacZ reporter mouse to measure transcriptional activity of CREB, Thome and colleagues found a more pronounced activating effect by the SSRI fluoxetine compared to the pro-noradrenergic antidepressant desipramine[17]. Imipramine, however, was not tested in this work.

The above mentioned studies investigated effects of antidepressants on CREB without inducing stress. In our study the most pronounced effect of imipramine was seen only in combination with chronic psychosocial stress where it exerts a reversal of the stress-induced CREB phsophorylation and activation. This finding parallels the clinical observation that antidepressants generally are not mood-elevating in healthy subjects but have anti-depressive effects when depression has become manifest. Thus it is tempting to speculate that the action of antidepressants on CREB strongly depends on its activity status and that the psychosocial stress generated in our model provides a necessary prerequisite for an effective action of antidepressants on CREB.

The notion that imipramine affects gene transcription through inhibition of CREB is also supported by the observation that various antidepressants down-regulate components of the cAMP signalling pathway, e.g. adenylate cyclase activity in the limbic forebrain[44]. Also, β-adrenergic receptor densitiy was decreased possibly due to reduced expression levels of the respective genes [45], [46]. Furthermore, tyrosine hydroxylase (TH), the rate-limiting enzyme of catecholamine biosynthesis showed diminished activity and newer findings indicate, that TH gene expression was also reduced after antidepressant treatment [47], [48]. All these genes contain CRE in their promoter region and are mainly regulated by CREB. As the chronic treatment with imipramine decreased CREB activity and phosphorylation, one may assume that CREB is the mediator of the antidepressant-induced adaptativ molecular processes.

In conclusion, our study contributes to the understanding of the molecular basis of depression and its treatment with antidepressants. On the basis of the data showing that the stress-induced CREB activation is attenuated by the antidepressant imipramine the controversial discussion on the action of antidepressants on CREB may be revisited. Our results support the view that antidepressants inhibit stress-induced CREB-directed transcription and thus strengthen the view that antidepressants induce neuronal adaptation on the transcriptional level.

## Materials and Methods

### Generation and analysis of transgenic mice

Transgenic mice were generated according to standard procedures. The 3.1 kb *BamH*I-*Apa*I fragment of 4xSomCRET81Luc[19] including four copies of the rat somatostatin gene promoter CRE (5′-GATCCTCCTTGGCTGACGTCAGAGAGAGAGTA-3′), truncated thymidine kinase promoter (−81 to−57) and the firefly luciferase-encoding gene (*Photinus pyralis*, Acc.No. M15077) (Fig. 1A) was gel-purified and microinjected into male pronuclei of fertilized eggs from NMRI mice. Microinjected eggs were transferred to the oviducts of foster mothers (CD1). Genomic (tail) DNA from the founder mice and offspring was analyzed by Southern blots. DNA was digested with *Bgl*II, electrophoresed, and immobilized on a Nylon membrane by capillary blotting. DNA was probed with a [α-^32^P]-labeled 1.6-kb *Xba*I luciferase gene fragment (Megaprime DNA labelling system, Amersham, Arlington Heights, IL, USA). Genomic tail DNA from offspring was analyzed by polymerase chain reaction with primers amplifying a 618-base pair fragment within the luciferase gene. All animal studies were conducted according to the National Institutes of Health's *Guidelines for Care and Use of Experimental Animals* and were approved by the Committee on Animal Care and Use of the local institution and state.

### Slice preparation

Transgenic mice were decapitated, brains were taken out and cut with a vibratome (Leica, Nussloch, Germany) into 300 µm coronal slices (interaural 6-0 according to Paxinos and Franklin[49]). Slices were treated with 10 µM forskolin (Sigma, Taufkirchen, Germany) for 6 h in carbogene-gassed incubation media containing 50% minimal essential medium, hanks' salts, 50% basal medium eagle, 2 mM glutamine (all from GibcoBRL, Karlsruhe, Germany) and 0,65% glucose, pH 7.36 at 37°C. Slices were shock-frozen in liquid nitrogen and stored at −80°C. For the luciferase assay tissue was homogenized in potassium phosphate buffer (0.1M K_2_HPO_4_, 0.1M KH_2_PO_4_ pH 7.8) supplemented with 1 mM DTT, 4 mM EGTA, 4 mM EDTA, 0.7 mM PMSF, 5 µg/ml leupeptin, 5 µg/ml pepstatin and 5 µg/ml aprotinin (all from sigma) and subjected to three cycles of freeze-thawing. After centrifugation 50 µl of supernatants were mixed with 370 µl assay buffer containing 16.5 mM potassium phosphate, 20 mM glycylglycine, 12 mM MgSO_4_, 3.2 mM EDTA, 1 mM DTT and 2.2 mM ATP (Sigma) and luciferase activity was measured by adding the substrate luciferin (Promega, Mannheim, Germany) in glycylglycine buffer (25 mM glycylglycine, 15 mM MgSO_4_, 4 mM EGTA, 10 mM DTT) with an automatic dispenser (Autolumat 953, Berthold, Bad Wildbach, Germany). Measured relative light units (RLU) were normalized to protein content determined by Bradford [50].

### Immunohistochemistry

Transgenic mice were anaesthetized and perfused intracardially via the left ventricle with 100 ml of 4% paraformaldehyde in PBS (0.1M phosphate buffer pH 7.2; 0.9% NaCl). Mice were decapitated and heads were post fixed in 4% paraformaldehyde over night. Brains were removed and washed over night in PBS following incubation in defrosting buffer (2% DMSO 20% glycerol in PBS) over night. Sagittal sections (50 µm) from interaural 0.5 to 1.5 mm [49] were cut using a cryostat and kept in PBS at 4°C. To inactivate endogenous peroxidases free floating sections were washed in 0.5% H_2_O_2_, permeabilized in 0.5% Triton X-100 and blocked with 5% normal rabbit serum in 0.5% TritonX-100 for 1 h. Sections were washed in 0.5% Triton X-100/PBS and incubated for 48 h at 4°C with anti-luciferase antibody (Promega, 4 µg/ml in blocking buffer). For detection of immunoreactivity peroxidase anti peroxidase (PAP) method was used described by Sternberger et al. [51]. Sections were washed intensively and than incubated with rabbit anti goat antibody (DAKO, Hamburg, Germany, diluted 1∶100) for 4 h. After washing, sections were incubated with anti-rabbit-PAP (DAKO, diluted 1∶100 in blocking buffer) for 2 h. Luciferase immunoreactivity was developed by incubating the sections for 5 min in 50 mM Tris pH 7.2 with 0.6 mg/ml of 3,3′-diaminobenzidine (DAB) and 0.3 mg/ml H_2_O_2_ (Vector Labs/Alexis Deutschland, Grünberg, Germany). After washing in 50 mM Tris pH 7.2 slices were mounted on gelatin-coated slides, dehydrated in ethanol/xylene and subjected to light microscopic inspection.

### Imaging of luciferase activity in cryostat sections

Transgenic mice were decapitated, brains were dissected and frozen immediately on liquid nitrogen. Sections (10–30 µm) were cut in a cryostat, thaw-mounted on slides and covered with luciferin substrate (Promega). Emitted photons were imaged for 10 min using a CCD camera system (Molecular Light Imager NightOwl, Berthold, Bad Wildbach, Germany).

### Dissection of brain regions

Transgenic mice were decapitated 14 h after the last treatment or stress exposure, brains were dissected and immediately frozen in liquid nitrogen. Brains were rinsed with icecold PBS and defined regions were dissected in the following order: Olfactory bulb, cerebellum, pons, frontal part of the brain anterior to the optic chiasm containing parts of the prefrontal cortex and the striatum (PFC), colliculi, hypothalamus, cortex and hippocampus. Pieces of tissue were frozen in liquid nitrogen and stored at −80°C. Luciferase activity was determined as described above.

### Western blot of CREB and phospho-CREB in homogenates

Transgenic mice were decapitated 14 h after the last stress exposure; brains were dissected on an ice-cold plate and immediately frozen in liquid nitrogen. For whole brain homogenates frozen tissue was pulverized under liquid nitrogen. Powder was transferred to boiling SDS-sample buffer and boiled for 5 minutes. For western blot of the different brain regions, regions were dissected on an ice-cold plate, transferred to boiling SDS-sample buffer, homogenized by a pistil and boiled again. Tissue lysates containing CREB/P-CREB were separated by 10% SDS-PAGE and transferred to nitrocellulose membrane by electroblotting. CREB and phospho-CREB were immunostained with specific antibodies (NewEngland Biolabs, Frankfurt a.M., Germany) and respective peroxidase-labeled secondary antibodies. Immunoreactivity was detected using the ECL reaction (Amersham Pharmacia, Germany). Specific bands were quantified densitometrically and the ratio between intensity of CREB and phospho-CREB from the same homogenate was calculated.

### Animal housing conditions

Animals were kept on a 12h- light-dark cycle. Food pellets and water were available *ad libitum*. For acute and chronic drug treatment mice were single-housed in plastic cages (28 cm L×16cm W×13 cm H).

### Stress model

The model of chronic psychosocial stress in mice [18] was established in our lab by Prof. Blanchard, University of Hawaii. For this experiment male transgenic mice (12–15 weeks old) were used. Two mice were housed in a cage separated by a perforated plastic partition. During 25 days the partition was removed every day for 10 min resulting in daily social conflicts. The daily interactions were recorded by two independent observers. After 3–5 days one mouse developed a dominant aggressive behaviour whereas the other became subordinate. Chasing and biting behavior was defined as dominant whereas flight, freezing and vocalization indicated the subordinate posture and thus social stress. Non-stressed mice from the same litter kept separately served as controls. Body weight of the subordinate, dominant and control animals was monitored throughout the experiment. On day 26, 14 h after the last stress exposure stressed and control mice were decapitated and brains were dissected.

### Antidepressant treatment

Acute treatment: 10 mg/kg imipramine HCl (Sigma) was applied i.p. at 9.00 h and 18.00 h. Mice were decapitated the following day at 8.00 h and brains were taken out for luciferase assay. Chronic treatment: 2×10 mg/kg/d imipramine HCl was applied i.p. for 21 days. The mice were decapitated on day 22 of the treatment at 8.00 h and brains were dissected. Chronic imipramine treatment in stressed mice: mice were exposed to chronic psychosocial stress. The submissive animal was treated from day 5 to day 25 with imipramine (2×10 mg/kg/d) while the daily stress procedure continued. On day 26, 14 h after the last stress exposure the mice were decapitated and brains were taken out. Within all treatments controls from the same litter received solvent only.

### Statistics

Data for *in vitro* stimulation of brain slices were not normal distributed and therefore analyzed by Wilcoxon-Test for paired samples (Fig 3c). The analysis of data for luciferase activity and CREB phosphorylation in brain regions was performed by two-factorial ANOVA (region vs. treatment) followed by Duncan's *post hoc* test (Fig. 4A and B, Fig.5 and table 2). Data for body weight gain was also analyzed with two-factorial ANOVA (time vs. status of the animals) followed also by Duncan's *post hoc* test (table 1). Western blot data were not normal distributed and therefore analyzed by non-parametrical ANOVA (Kruskal-Wallis-Test, followed by Schaich-Hamerle *post hoc* test (Fig. 6). All analyses were performed using the soft ware STATISTICA 7.1, statsoft, Tulsa, USA.
